# Comparison of masticatory performance and tongue pressure between children and young adults

**DOI:** 10.1002/cre2.104

**Published:** 2018-03-22

**Authors:** Yuko Fujita, Maika Ichikawa, Ayako Hamaguchi, Kenshi Maki

**Affiliations:** ^1^ Division of Developmental Stomatognathic Function Science, Department of Health Promotion Kyushu Dental University Japan

**Keywords:** BMI, DMFT index, masticatory performance, schoolchildren, tongue pressure, young adults

## Abstract

The aims of the present study were to evaluate whether there are significant differences in masticatory performance by gender and dental stage. We also determined the factors directly associated with the masticatory performance in children, and those directly associated with masticatory performance in young adults. The study included 180 subjects, ranging in age from 6 to 12 years or 20 to 33 years. The subjects were divided into three groups according to the Hellman developmental stage (III A, III B, or VA); the groups were the subdivided according to gender. The body mass index (BMI), maximum tongue pressure, and sum of decayed, missing, and filled teeth (DMFT) were determined in all subjects. To investigate masticatory performance, the total number and maximum projected area of chewed particles of the jelly materials were measured. Masticatory performance had the highest values at Stage VA in both males and females. Regarding the maximum tongue pressure in females, Stage III B had the highest value of all stages. Multiple regression analysis showed that masticatory performance was associated with DMFT index, maximum tongue pressure, and BMI in children. Among young adults, masticatory performance was associated with DMFT index and maximum tongue pressure. Better masticatory performance is directly associated with better dental status, a higher BMI, and tongue pressure in schoolchildren. Additionally, masticatory performance was well‐correlated with tongue pressure in young adults, although maximum tongue pressure reached its peak before Stage VA in females. We suggest that females need training with respect to tongue pressure, by the mixed dentition stage.

## 1 INTRODUCTION

Clinical studies have reported that masticatory performance increases during childhood (Toro, Buschang, Throckmorton, & Roldan, 2006) and adolescence and declines with age in older adults (Peyron, Blanc, Lund, & Woda, 2004). We believe that masticatory performance peaks in young adulthood and then plateaus and finally declines as a natural maturation phenomenon, although maintaining an adequate number of healthy natural teeth helps maintain adequate masticatory performance in the elderly (Ikebe et al., 2011). We postulated that, to inhibit any decrease in masticatory performance, it is important to maintain it at a level equal to its peak level, and it is also important to attain as high a peak masticatory performance as possible. In humans, after 2–4 years of age, the tongue is used during mastication because the mature swallowing pattern prevails over the infantile swallowing pattern (Peng, Jost‐Brinkmann, Yoshida, Chou, & Lin, 2004). There have been a number of reports concerning the relationship between tongue pressure and chewing ability in young adults (Takahashi, Koide, Arakawa, & Mizuhashi, 2013; Yamada, Kanazawa, Komagamine, & Minakuchi, 2015). Additionally, studies on the relationship between chewing ability and indices of physical constitution, such as body mass index (BMI) and body weight in children with primary dentition, were also reported (Consolacao Soares et al., 2017; Soares et al., 2017). However, there are few reports comparing the degree of association of masticatory performance with tongue pressure and BMI between children with mixed dentition and young adults.

Studies have suggested that the number and area of occlusal contacts influence masticatory performance (English, Buschang, & Throckmorton, 2002; Gibbs, Lundeen, Mahan, & Fujimoto, 1981). In schoolchildren, the growth stage of the dentition varies significantly from 6 to 12 years of age, and masticatory performance in these children may be influenced by missing deciduous teeth. Therefore, this study focused on the dental development stage to determine masticatory performance and tongue pressure.

This study evaluated whether there are significant differences in masticatory performance and tongue pressure by gender and dental stage. We also determined the factors directly associated with the masticatory performance in children and those directly associated with masticatory performance in young adults.

## 2 MATERIALS AND METHODS

### 2.1 Participants

This study was approved by the Human Investigations Committee of Kyushu Dental University (Approval Number 16‐27), and all subjects provided written informed consent prior to participation. For the children, we explained this study clearly depending on the degree of understanding of each child. After the children had agreed to participate, we obtained their parents' consent.

We planned a study in which regression of the total number of chewed particles of jelly against the subjects' decayed, missing, and filled teeth (DMFT) index would be performed. Previously obtained data indicated that the standard deviation of the total number of particles is 20 and the standard deviation of the regression errors is 18.33 among subjects without this examination (Ichikawa, Fujita, Hamaguchi, Chaweewannakorn, & Maki, 2016). If the true slope of the line obtained by regressing the total number of particle against the DMFT index is −0.5, a minimum sample of 28 subjects in each group is needed to achieve 80% power at a two‐sided 5% significance level. Based on these predicted data, the sample consisted of 120 healthy subjects ranging in age from 6 to 12 years (60 males and 60 females) and 60 healthy young adults aged from 20 to 33 years (30 males and 30 females). The subjects were recruited following an initial examination at Kyushu Dental University Hospital and divided into the following three groups (60 per group) based on the Hellman developmental stage: complete eruption of permanent first molar or incisors (III A); exchange phase of lateral teeth (III B); and complete eruption of permanent third molar (VA). The third molars of all subjects had been extracted. Each group was further subdivided by gender (*n* = 30 for each subgroup).

The exclusion criteria were systemic disturbances, ingestion of medicines that could interfere directly or indirectly with muscular activity, and uncooperative behavior. In addition, children and adults with alterations in the form, structure, or number of teeth or oral tissues were excluded, as were those with a history of orthodontic treatment or temporomandibular dysfunction (Castelo, Gaviao, Pereira, & Bonjardim, 2005).

### 2.2 Anthropometry and dental examination

Measurements of height and body weight were taken in the consulting room of the hospital. Height was measured to an accuracy of ±0.1 cm using a portable digital stadiometer (AD‐6531; A&D, Tokyo, Japan) with the head in the Frankfort plane and body weight to within 0.1 kg. The BMI was calculated according to the formula, BMI = kg/m^2^. To evaluate the severity of obesity, BMI *z*‐scores and BMI classified based on World Health Organization criteria were used in children and young adults, respectively (de Onis et al., 2007; World Health Organization, 2000).

During the intraoral examination, DMFT was calculated using criteria recommended by the World Health Organization (World Health Organization, 2013).

### 2.3 Maximum tongue pressure

Maximum tongue pressure was measured using a tongue pressure manometer (JMS, Hiroshima, Japan). Subjects were examined in a relaxed sitting position, in which the Frankfort plane was maintained horizontal. Subjects were asked to place a balloon on the anterior part of their palate and to close their lips, biting a hard ring with the upper and lower incisors. Then, the subjects were asked to raise their tongues and compress the balloon onto the palate with maximal voluntary muscular effort for approximately 7 s. The pressure was measured (in kilopascals) using a digital voltmeter attached to the tongue pressure manometer (Takahashi et al., 2013).

### 2.4 Masticatory performance

Masticatory performance was evaluated by determining each individual's ability to comminute a jelly‐based chewable material (Kamuzokun®; Mamarisshimo, Tokyo, Japan). The chewable samples had dimensions of 15 × 15 × 15 mm and consisted of maltitol, gelatin, sweetener (xylitol), and thickener (Arabian gum). Before the experiments, the children were shown how to perform the masticatory movements, as well as the mouth‐rinsing procedure, to ensure that they would not swallow.

In line with the manufacturer's instructions, after chewing the jelly‐based samples for 60 s, the subjects were instructed to stop chewing, expectorate the sample into a plastic filter, and rinse with water until all particles were removed from the mouth. The chewed particles were then washed with water and dried at room temperature on filter paper (Super Absorption Paper®; Mamarisshimo, Tokyo, Japan) for 10 min. The samples were placed on white filter papers, and a digital camera (D750; Nikon, Tokyo, Japan) was used to photograph the samples from above under standard lighting. A plastic calibration ruler was used as an index of length. ImageJ software (National Institutes of Health, Bethesda, MD, USA) was used to measure the total number and maximum projected area (in units of mm^2^) of the particles (Ichikawa et al., 2016; Ikebe et al., 2011).

### 2.5 Reliability of measurements

All measurements were duplicated and separated by a 30‐s rest period, and the mean values were used in the analysis. In addition, the total number of particles and maximum projected area were measured twice according to an interval of 2 weeks, and the mean values were used in the analysis (Ichikawa et al., 2016). All examinations were carried out by the same trained examiner. The data generated during the examinations were assessed for reliability. Random error was characterized based on the intrarater reliability, which was quantified using the intraclass correlation coefficient (ICC). The results of the test–retest reliability were expressed in terms of ICC: 0.900 ≤ ICC ≤ 1.000 corresponded to excellent reliability (Domholdt, 1993).

### 2.6 Data analysis

The data for each age group are presented as means ± standard deviation (SD). Multiple comparisons among groups were done using one‐way analysis of variance with Tukey's honest significant difference test. Pearson's or Spearman's correlation coefficients were used to determine the associations among variables. Gender was coded as 1 for male and 0 for female. The total number of particles was assessed as reflecting masticatory performance in the multiple linear regression analysis. Stepwise multiple linear regression analysis was used to investigate the relationship between the total number of particles (dependent variable) and all parameters that showed significant associations in Pearson's or Spearman's correlation analyses (independent variables), where *p* < .05 was considered to indicate statistical significance. All data were analyzed using SPSS for Windows (ver. 23.0; IBM Japan, Tokyo, Japan).

## 3 RESULTS

The ICCs for height, body weight, DMFT index, maximum tongue pressure, total number of chewed particles, and maximum projected area of the particles were all ≥0.95.

The prevalence of obese and overweight among the children was 14.2% (*n* = 17), with the remaining 103 children (85.8%) being the normal range. Among the young adults, the prevalence of overweight was 15.0% (*n* = 9), with the remaining 51 young adults (85.0%) being in the normal range.

Table 1 lists the means (±SD) of the anthropometric measurements and the DMFT index. Males and females in the VA group had significantly a higher DMFT index than the females in the III B group.

**Table 1:** Anthropometric parameters and DMFT index by Hellman stage and gender

Hellman's stages	Gender	*N*	Age (years)	Height (cm)	Body weight (kg)	BMI	DMFT index
III A	M	30	7.00 ± 0.830	126.500 ± 6.257	27.343 ± 5.476	16.921 ± 2.091	2.50 ± 2.316
	F	30	6.97 ± 0.809	125.000 ± 6.930	24.867 ± 3.697	15.835 ± 1.353	2.83 ± 2.829
III B	M	30	10.03 ± 0.964	140.410 ± 7.624^a^, ^b^	35.203 ± 5.321	17.866 ± 2.777^b^	2.67 ± 2.644
	F	30	10.00 ± 0.947	141.077 ± 7.682^a^, ^b^	36.497 ± 6.037^a^, ^b^	18.349 ± 3.042^b^	1.60 ± 1.653
VA	M	30	25.30 ± 3.239	171.547 ± 5.498^a^, ^b^, ^c^, ^d^	69.187 ± 8.319^a^, ^b^, ^c^, ^d^	23.540 ± 2.895^a^, ^b^, ^c^, ^d^	4.07 ± 4.076^d^
	F	30	25.90 ± 2.695	159.017 ± 5.943^a^, ^b^, ^c^, ^d^, ^e^	49.283 ± 6.774^a^, ^b^, ^c^, ^d^, ^e^	19.539 ± 2.975^a^, ^b^, ^e^	4.37 ± 2.632^d^

*Note*. Data are expressed as means ± standard deviation. III A = complete eruption of permanent first molar or incisors; III B = exchange phase of lateral teeth; VA = complete eruption of permanent third molars; M = male; F = female. BMI = body mass index; DMFT = decayed, missing, and filled teeth.

The significant difference was defined by one‐way analysis of variance and Tukey's honest significant difference test.

a *p* < .05 versus the III A male group.

b *p* < .05 versus the III A female group.

c *p* < .05 versus the III B male group.

d *p* < .05 versus the III B female group.

e *p* < .05 versus the VA male group.

Table 2 lists the means (±SD) for maximum tongue pressure and masticatory performance. Regarding maximum tongue pressure among males, the VA group showed the highest value among all groups of males. On the other hand, in females, the III B group showed the highest value among all groups. In the VA group only, a statistically significant difference in maximum tongue pressure was found between males and females (*p* < .05). Regarding the total number of particles, values in the III B female, VA male, and VA female groups were significantly higher than those in the III A male, III A female, and III B male groups (*p* < .05). Regarding the maximum projected area of the particles, values in the VA male and VA female groups were significantly lower than those in the III A male, III A female, III B male, and III B female groups (*p* < .05).

**Table 2:** Maximum tongue pressure, total number of particles, and maximum projected area of the particles by Hellman stage and gender

Hellman's stages	Gender	Maximum tongue pressure (kPa)	Total number of particles (*N*)	Maximum projected area of the particles (mm^2^)
III A	M	30.117 ± 6.750	40.33 ± 24.632	77.083 ± 40.092
	F	30.553 ± 4.813	39.63 ± 20.054	70.564 ± 15.344
III B	M	33.300 ± 7.170	40.10 ± 21.305	82.994 ± 39.639
	F	36.877 ± 3.889^a^ b	57.97 ± 21.159^a^, ^b^, ^c^	65.741 ± 23.022
VA	M	39.627 ± 4.127^a^, ^b^, ^c^	72.63 ± 23.605^a^, ^b^, ^c^	38.479 ± 9.251^a^, ^b^, ^c^, ^d^
	F	30.503 ± 4.683^d^, ^e^	65.17 ± 12.573^a^, ^b^, ^c^	41.717 ± 5.412^a^, ^b^, ^c^, ^d^

*Note*. Data are expressed as means ± standard deviation. III A = complete eruption of permanent first molar or incisors; III B = exchange phase of lateral teeth; VA = complete eruption of permanent third molars; M = male; F = female.

The significant difference was defined by one‐way analysis of variance and Tukey's honest significant difference test.

a *p* < .05 versus the III A male group.

b *p* < .05 versus the III A female group.

c *p* < .05 versus the III B male group.

d *p* < .05 versus the III B female group.

e *p* < .05 versus the VA male group.

Table 3 lists the results of the Pearson's and Spearman's bivariate correlation analyses for the sample of children. The total number of particles was significantly correlated with age, gender, body weight, BMI, DMFT index, maximum tongue pressure, and maximum projected area of the particles. There was a strong correlation between the total number of particles and the maximum projected area of the particles in the children (*r* = −.758, *p* < .001).

**Table 3:** Bivariate correlation coefficients among anthropometric parameters, DMFT index, maximum tongue pressure, and masticatory performance in children

	Age	Gender	Height	Body weight	BMI	DMFT index	Maximum tongue pressure	Total number of particles	Maximum projected area of the particles
Age	1								
Gender	0.010	1							
Height	0.852**	0.033	1						
Body weight	0.761**	0.066	0.823**	1					
BMI	0.343**	0.105	0.272**	0.763**	1				
DMFT index	−0.049	0.063	−0.045	−0.116	−0.150	1			
Maximum tongue pressure	0.403**	−0.133	0.232*	0.306**	0.257**	−0.580**	1		
Total number of particles	0.249**	−0.182*	0.171	0.318**	0.350**	−0.773**	0.693**	1	
Maximum projected area of the particles	−0.124	0.133	−0.086	−0.178	−0.214*	0.694**	−0.664**	−0.758**	1

*Note*. DMFT = decayed, missing, and filled teeth; BMI = body mass index.

Gender was coded as 1 for male and 0 for female.

* *p* < .05.

** *p* < .01.

Table 4 shows the results of the stepwise linear regression analysis done to identify independent variables that significantly contributed to variation in the total number of particles. After adjusting for collinearity among the independent variables, the stepwise linear regression model identified that the total number of particles was significantly associated with the DMFT index (*β*: standardized partial regression coefficient = −0.559, *p* < .001), maximum tongue pressure (*β* = 0.321, *p* < .001), and BMI (*β* = 0.184, *p* < .001).

**Table 4:** Stepwise multiple linear regression analysis of total number of particles in children

Dependent variable	Independent variables	B	SE	*β*	*p* value
Total number of particles	DMFT index	−5.301	0.573	−0.559	<.001
	Maximum tongue pressure	1.162	0.223	0.321	<.001
	BMI	1.644	0.456	0.184	<.001

*Note*. DMFT = decayed, missing, and filled teeth; BMI = body mass index; B = unstandardized partial regression coefficient; SE = standard error*; β =* standardized partial regression coefficient indicating the relative importance of each variable.

*R* = .848, *R* ^2^ = .719, adjusted *R* ^2^ = .712, *p* < .001.

Table 5 lists the results of the Pearson's and Spearman's bivariate correlation analyses for the sample of young adults. The total number of particles was significantly correlated with gender, DMFT index, maximum tongue pressure, and maximum projected area of the particles. There was a strong correlation between the total number of particles and the maximum projected area of the particles in the young adults (*r* = −.837, *p* < .001).

**Table 5:** Bivariate correlation coefficients among anthropometric parameters, DMFT index, maximum tongue pressure, and masticatory performance in young adults

	Age	Gender	Height	Body weight	BMI	DMFT index	Maximum tongue pressure	Total number of particles	Maximum projected area of the particles
Age	1								
Gender	−0.320*	1							
Height	−0.289*	0.765**	1						
Body weight	−0.144	0.831**	0.673**	1					
BMI	−0.026	0.647**	0.244	0.878**	1				
DMFT index	0.307*	−0.114	−0.070	−0.042	−0.016	1			
Maximum tongue pressure	−0.255*	0.704**	0.549**	0.599**	0.410**	−0.313*	1		
Total number of particles	−0.201	0.336**	0.158	0.137	0.066	−0.615**	0.542**	1	
Maximum projected area of the particles	0.175	−0.364**	−0.165	−0.260*	−0.212	0.661**	−0.624**	−0.837**	1

*Note*. BMI = body mass index; DMFT = decayed, missing, and filled teeth.

Gender was coded as 1 for male and 0 for female.

* *p* < .05.

** *p* < .01.

Table 6 shows the results of the stepwise linear regression analysis done to identify independent variables that significantly contributed to variation in the total number of particles. After adjusting for collinearity among the independent variables, the stepwise linear regression model identified that the total number of particles was significantly associated with the DMFT index (*β* = −0.494, *p* < .001) and maximum tongue pressure (*β* = 0.387, *p* < .001).

**Table 6:** Stepwise multiple linear regression analysis of total number of particles in young adults

Dependent variable	Independent variables	B	SE	*β*	*p* value
Total number of particles	DMFT index	−2.774	0.546	−0.494	<.001
	Maximum tongue pressure	1.165	0.293	0.387	<.001

*Note*. DMFT = decayed, missing, and filled teeth; B = unstandardized partial regression coefficient; SE = standard erro*r; β =* standardized partial regression coefficient indicating the relative importance of each variable.

*R* = .717, *R* ^2^ = .513, adjusted *R* ^2^ = .496, *p* < .001.

## 4 DISCUSSION

We found a strong correlation between the total number of particles and the maximum projected area of the particles of chewed jelly in both the children and the young adults; in addition, the intrarater reliability was high. These results suggest that our method for measuring masticatory performance was useful quantitatively. In addition, masticatory performance in males and females had the highest value at Stage VA. There were some variances in the maximum projected area of the particles in children with mixed dentition, and their parameters of masticatory performance did not show statistically significant differences among all groups, except for the total number of particles in the III B female group. These results suggest that masticatory performance did not change significantly during the mixed dentition stage, and masticatory performance was facilitated at or after stage III B.

In recent years, various methods for characterizing masticatory performance have been reported, including a method that calculates the median particle size of an artificial silicone test food (Eberhard et al., 2012; Soares et al., 2017). It is possible to apply that technique with jelly materials. However, we selected a method that allows visual observation of the total number of the particles and the maximum projected area of the particles of chewed jelly, simplifying the evaluation.

Based on masticatory performance measured by the total number of particles, the present study showed that a lower DMFT index, higher tongue pressure, and higher BMI were associated with a higher masticatory performance in children with mixed dentition. A lower DMFT index and higher tongue pressure were associated with a higher masticatory performance among the young adults. DMFT index was the variable most strongly associated with masticatory performance in both the children and young adults. These results were consistent with previous studies of 8–12‐year‐old children (de Souza Barbosa, de Morais Tureli, Nobre‐dos‐Santos, Puppin‐Rontani, & Gaviao, 2013; de Morais Tureli, de Souza Barbosa, & Gaviao, 2010).

In this study, masticatory performance was directly correlated with BMI in children. Previous studies also indicated that body size was the variable most strongly associated with the masticatory performance of children (Julien, Buschang, Throckmorton, & Dechow, 1996; Toro et al., 2006). In contrast, some studies reported that poorer masticatory performance was associated with a higher BMI in 3–5‐year‐old children (Consolacao Soares et al., 2017; Soares et al., 2017). However, they also reported that a low chewing ability was associated with a greater frequency of daily ingestion of liquid foods among children with a higher BMI (Consolacao Soares et al., 2017). Another study suggested that caloric compensation was consistently poorer in heavier children age 4–5 years due to a preference for junk foods among the overweight/obese children (Carnell, Benson, Gibson, Mais, & Warkentin, 2017). These findings indicate that poor dietary habits indulged by parents, rather than skeletal growth, caused the high BMI of the 3–5‐year‐old children in these studies. We suppose that the higher BMI in children with mixed dentition was accompanied by the growth of bones and muscles, when their nutritional condition and energy expenditure were well‐controlled by school lunch and physical exercise in school life (Asakura & Sasaki, 2017; Tamaki et al., 2008).

Another study found that no gender differences in the masticatory performance of 6–15‐year‐old children and adolescents (Toro et al., 2006). In our study, the masticatory performance of Stage III B females was superior to that of stage‐matched males; this may have been due to the differences in BMI, DMFT index, and maximum tongue pressure between females and males in Stage III B.

In this study, maximum tongue pressure was also well‐correlated with masticatory performance, both in the children and young adults. In the recent studies, tongue pressure and masticatory performance were positively correlated in a sample of young adults (Takahashi et al., 2013); additionally, significant differences in maximum tongue pressure were found between males and females in young adults (Takahashi et al., 2013; Vanderwegen, Guns, Van Nuffelen, Elen, & De Bodt, 2013). The findings were consistent with their results. Pitts, Stierwalt, Hageman, and LaPointe (2017) reported that there was a positive correlation between palatal width and maximum tongue pressure in adult patients. The differences in maximum tongue pressure between gender and dental age may have been due to the differences in the size of palatal bone.

Interestingly, we found that the peak maximum tongue pressure occurred earlier in females compared with males. The maximum tongue pressure in females had the highest value at Stage III B. Utanohara et al. (2008) reported that the mean maximum tongue pressure was around 35 kPa among Japanese females aged 20–30 years. In this study, the mean maximum tongue pressure in Stage VA females was 30.503 kPa, although that in Stage III B females was 36.877 kPa. These different results may have been due to the differences in measurement device and the differences in body size (including palatal bone size) of the participants.

Several studies showed that maximum tongue pressure remained constant from 20s to 60s and started to decrease from the 70s in females (Utanohara et al., 2008; Vanderwegen et al., 2013). We suggest that females need training with respect to tongue pressure, by the mixed dentition stage, to increase the peak of tongue pressure. Hiramatsu, Kataoka, Osaki, and Hagino (2015) reported a mean maximum tongue pressure, of men and women over the age of 70 years, of 26.85 ± 0.68 kPa. These findings suggest that maximum tongue pressure in males dramatically decreases, relative to females, after the peak period.

## 5 CONCLUSION

We demonstrated that superior masticatory performance is directly associated with better dental status and a higher BMI and tongue pressure among children. Additionally, better dental status and higher tongue pressure also contribute to superior masticatory performance in young adults. However, maximum tongue pressure reached its peak before Stage VA in females. We suggest that females need training with respect to tongue pressure, by the mixed dentition stage.
